# Supplementary material for: Valley-polarized domain wall magnons in 2D ferromagnetic bilayers
Source: Sci Rep. 2020 Oct 7;10:16733. doi: 10.1038/s41598-020-74047-3 (PMC7542437; doi:10.1038/s41598-020-74047-3)
Supplement: Supplementary file 1 — Supplementary Information. [file 41598_2020_74047_MOESM1_ESM.pdf]

Supplementary Information

Valley-polarized domain wall magnons in 2D ferromagnetic bilayers

D. Ghader

Supplementary Note. Derivation of FBL magnonic Hamiltonian.

An A-site in the honeycomb lattice has three nearest neighbors with relative position vectors $\vec{\delta}_1^A = a(0, 1/\sqrt{3})$, $\vec{\delta}_2^A = a(1/2, -\sqrt{3}/6)$, and $\vec{\delta}_3^A = a(-1/2, -\sqrt{3}/6)$. The lattice constant a denotes the $A - A$ (or $B - B$) distance. The 6 next nearest neighbors vectors for both A and B sublattices are $\vec{\gamma}_1 = a(1/2, -\sqrt{3}/2), \vec{\gamma}_2 = a(-1/2, -\sqrt{3}/2), \vec{\gamma}_3 = a(1, 0), \vec{\gamma}_4 = -\vec{\gamma}_1, \vec{\gamma}_5 = -\vec{\gamma}_2$, and $\vec{\gamma}_6 = -\vec{\gamma}_3$. Vectors $\vec{\delta}_i^A$ and $\vec{\gamma}_j$ are illustrated schematically in Fig.S1.

The real space Heisenberg Hamiltonian for AB-stacked FBL with nearest neighbor exchange, DMI, and ED reads

$$\mathcal{H}_{AB} = -J \sum_{l,\vec{\delta}_i^A} \vec{S}^{A_l}(\vec{R}_{A_l}, t).\vec{S}^{B_l}(\vec{R}_{A_l} + \vec{\delta}_i^A, t) - J_\perp \sum_{\vec{R}_{A_1}} \vec{S}^{A_1}(\vec{R}_{A_1}, t).\vec{S}^{B_2}(\vec{R}_{A_1} + \vec{\delta}_\perp, t)$$

$$+ \sum_{\alpha,l,\vec{\gamma}_j} D_z(\vec{R}_{\alpha_l}, \vec{R}_{\alpha_l} + \vec{\gamma}_j)\vec{S}^{\alpha_l}(\vec{R}_{\alpha_l}, t).\vec{S}_D^{\alpha_l}(\vec{R}_{\alpha_l} + \vec{\gamma}_j, t) - \sum_{\alpha,l} \Delta_l \hat{z}.\vec{S}^{\alpha_l}(\vec{R}_{\alpha_l}, t) \tag{S1}$$

J and $J_\perp$ are the nearest neighbor in-plane and interlayer exchange coefficients respectively. The vector $\vec{\delta}_\perp$ connects $A_1 - B_2$ dimer sites. The alternating next nearest neighbor DMI vector has the form $\vec{D}(\vec{r}, \vec{r} + \vec{\gamma}_j) = D_z\hat{z} = \pm D\hat{z}$, where the orientation of $\vec{D}$ is determined in the conventional way from the local geometry of the honeycomb lattice [5]. Index α stands for A or B sublattices while index l specifies the layer ($l = 1, 2$). $\vec{S}^{\alpha_l}(\vec{R}_{\alpha_l}, t)$ is the spin on site $\vec{R}_{\alpha_l}$ at time t. We have also introduced $\vec{S}_D^{\alpha_l} = S_y^{\alpha_l}\,\hat{x} - S_x^{\alpha_l}\,\hat{y}$ to express the DMI term in the form of a scalar-product rather than a cross-product [21]. The ED potentials are denoted $\Delta_l = \pm\Delta$ for $l = 1, 2$ respectively.

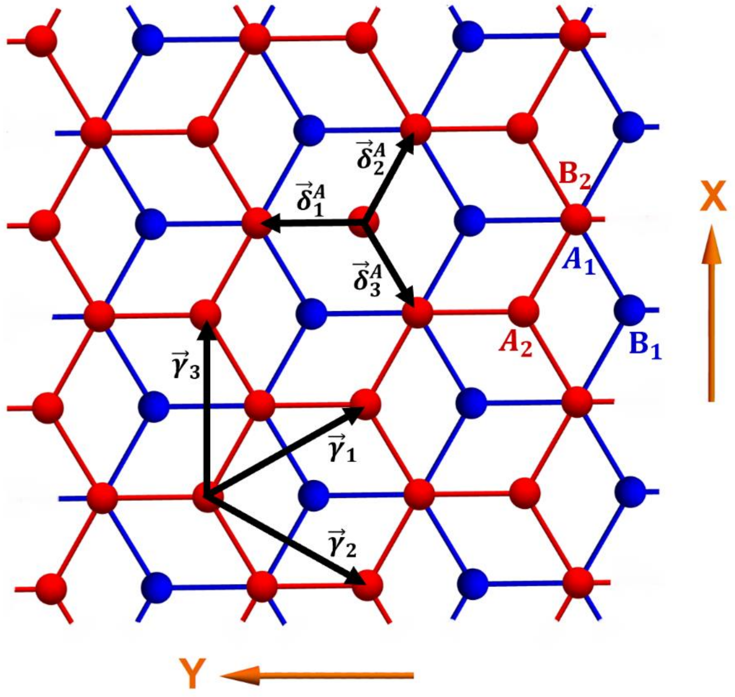

Figure S1: Schematic representation showing a top view of the AB-stacked honeycomb FBL. The figure also presents the relative position vectors for the nearest and next nearest neighbors of an A-site. Figure generated using Mathematica Software version 12 https://www.wolfram.com/mathematica/

In what follows, we adopt the semi-classical linear spin wave approach [18-22], where the spins $\vec{S}^{\alpha_l}$ are treated as numerical vectors. The spin dynamics are determined using the Landau-Lifshitz (LL) equations of motion. We start with the effective field acting on the A_1 −sublattice, deduced from $\mathcal{H}_{AB}$ as

$$\vec{H}^{A_1}(\vec{R}_{A_1}, t) = -J_\perp \vec{S}^{B_2}(\vec{R}_{A_1} + \vec{\delta}_\perp, t) + \Delta\,\hat{z} - J \sum_{\vec{\delta}_i^A} \vec{S}^{B_1}(\vec{R}_{A_1} + \vec{\delta}_i^A, t) + \sum_{\vec{\gamma}_j} D_z(\vec{R}_{A_1}, \vec{R}_{A_1} + \vec{\gamma}_j) \vec{S}_D^{A_1}(\vec{R}_{A_1} + \vec{\gamma}_j, t) \tag{S2}$$

The LL equation of motion of A_1 site, $\partial_t \vec{S}^{A_1} = \vec{S}^{A_1} \times \vec{H}^{A_1}$, keeping only linear terms yields

$$i\partial_t S^{A_1}(\vec{R}_{A_1}, t) = (3JS + J_\perp S + \Delta) S^{A_1}(\vec{R}_{A_1}, t) - JS \sum_{\vec{\delta}_i^A} S^{B_1}(\vec{R}_{A_1} + \vec{\delta}_i^A, t) - SJ_\perp S^{B_2}(\vec{R}_{A_1} + \vec{\delta}_\perp, t) - iS \sum_{\vec{\gamma}_j} D_z(\vec{R}_{A_1}, \vec{R}_{A_1} + \vec{\gamma}_j) S^{A_1}(\vec{R}_{A_1} + \vec{\gamma}_j, t) \tag{S3}$$

with $S^{\alpha_l} = S_x^{\alpha_l} - iS_y^{\alpha_l}$. The symbol S denotes the constant z component of the spin vector.

Next, Fourier transformation is applied to Eq. S3 to determine the momentum-space version of the equation of motion

$$i\partial_t S^{A_1}(\vec{p},t) = [3JS + J_\perp S + \Delta + DS\, h(\vec{p})]S^{A_1}(\vec{p},t) - JSf(\vec{p})S^{B_1}(\vec{p},t) - SJ_\perp S^{B_2}(\vec{p},t) \tag{S4}$$

with $f(\vec{p}) = e^{ip_y\frac{a}{\sqrt{3}}} + 2e^{-i\frac{\sqrt{3}a}{6}p_y}\cos\left(\frac{a}{2}p_x\right)$, $h(\vec{p}) = 4sin\left(\frac{a}{2}p_x\right)\cos\left(\frac{\sqrt{3}a}{2}p_y\right) - 2sin(p_x a)$. p_x and p_y are the momenta along x and y directions.

Equations for S^{B_1}, S^{A_2} and S^{B_2} can be derived in a similar manner. Collecting the four momentum-space equations of motion results in a Schrödinger matrix equation

$$i\partial_t|\Psi(\vec{p})\rangle = \mathcal{H}_{AB}(\vec{p})|\Psi(\vec{p})\rangle \tag{S5a}$$

with the 4-band momentum-space Hamiltonian

$$\mathcal{H}_{AB}(\vec{p}) = JS\begin{pmatrix}(3+U)I + \mathcal{H}_1 & \mathcal{H}_3 \\ \mathcal{H}_3^\dagger & (3-U)I + \mathcal{H}_2\end{pmatrix} \tag{S5b}$$

In Eq. S5b, we have defined

$$\mathcal{H}_1 = \begin{pmatrix} f_D(\vec{p}) + v_0 & -f(\vec{p}) \\ -f^*(\vec{p}) & -f_D(\vec{p})\end{pmatrix}, \mathcal{H}_2 = \begin{pmatrix} f_D(\vec{p}) & -f(\vec{p}) \\ -f^*(\vec{p}) & -f_D(\vec{p}) + v_0\end{pmatrix}, \mathcal{H}_3 = \begin{pmatrix} 0 & -v_0 \\ 0 & 0\end{pmatrix}, I = \begin{pmatrix} 1 & 0 \\ 0 & 1\end{pmatrix},$$

$v_0 = J_\perp/J$, $f_D(\vec{p}) = D\, h(\vec{p})\,/\,J$ and $U = \Delta/JS$.
